# The Vaccination Question

**Published:** 1896-05-16

**Authors:** Arthur Wollaston Hutton

**Affiliations:** Chiswick


### Editor's Letter-Box.

[Our correspondents are reminded that prolixity is a great bar to publication, and that brevity of style and conciseness of statement greatly facilitate early insertion.]

### THE VACCINATION QUESTION.

Sir,—Having only just returned after six months' absence on a visit to New Zealand, I have not until to-day been able to read the review of my "Vaccination Question," which appeared in your columns on April 4th. Will you allow me to comment on one or two points in it?

Your reviewer complains that I add nothing to what has already been said by "those competent anti-vaccinatists, Creighton and Crookshank." Well, I do give sundry illustrated details as to the failure of vaccination to prevent small-pox, which were outside the purview of both those writers; but in the main I admit it was my object to "rub in" what they already had said. The average layman takes his medical doctrine from his doctor with as much docility as the average Irish Catholic takes his theological doctrine from his priest. But on the rare occasions when a layman does wake up and give attention to this subject, he finds in Creighton and Crookshank so solid a scientific (pathological) case against vaccination, that it really would not be worth my while to attempt to add anything to it, especially as no medical man has replied to their argument, though it has been before the profession eight or nine years.

Secondly, your reviewer says that if I and other anti-vaccinators "would frankly admit that the facts as to the value of vaccination are absolutely against us," the public would be more ready to reconsider the question of compulsion. Surely this is absurd on the face of it. If the public reconsider the question of compulsion, it will not be because all men have been brought round to confess that vaccination is a good thing, but because it has come to realise that the risks involved in vaccination are less doubtful than the advantages claimed for it. What your reviewer really means is, "if these men will humbly bow their heads before us, the medical profession, we shall feel disposed to deal graciously with them; but we will grant them no indulgence while they remain contumacious." Now this is the old sacerdotal arrogance over again. "Make your submission, hypocrital it may be—it certainly would be in this case—and we will then show you favour." That the statistics, as issued, are almost uniformly against us, we of course admit; but that they can be a true representation of the facts we deny, and must deny, so long as the pathological case against vaccination remains unrefuted.

Medical men now very commonly admit in private conversation that they think vaccination should not be enforced by law; but they will not raise a little finger to get rid of the enforcement; though the thing would be done at once if a deputation of medical men—a really representative deputation—took the matter in hand. They do not realise how injurious to the best interests of their profession is the existence of the compulsory law. I am speaking now not as an "anti-vaccinator" but as an observer of human affairs; and I am confident that in these days, when every doctrine or practice, however sacred or well established, is examined, criticised, and perhaps condemned by eager, intelligent, and —if you will—captious men, the medical profession can no more afford to have a prescription enforced by law than the clergy could afford to have baptism or the communion similarly enforced, admirable prophylactics against spiritual evil as both those institutions are generally believed to be. The vaccination law differs from all other measures enforcing sanitary precautions. It is in effect a Test Act, involving a profession of faith, and, as such, it ought to be repealed.—I am, sir, your obedient servant,

Arthur Wollaston Hutton.

Chiswick, May 7th, 1896.

